# Annotations

**Published:** 1894-03-17

**Authors:** 


### ANNOTATIONS.

### Headaches.

Dr. Lauder Brunton has recently published a paper on headaches, some of the conclusions of which may be of practical interest during the present "muggy" weather when headaches are so extensively prevalent. What are the most common of all causes of headache? "Visual defects," according to Dr. Brunton. Between 80 and 90 per cent. of all headaches he considers to be due to these defects. Therein, perhaps, his generalisation is open to a little criticism. Decayed teeth, too, play a prominent role in some headaches. Dr. Brunton holds that as many as 10 per cent. of all headaches are due to this cause. The remaining 5 per cent. he believes to be produced by diseases of the nose and other casual circumstances. This generalisation seems to us to be too sweeping, and if space permitted we think good reasons could be adduced, apart from the eyes or the teeth, why headaches in our large town climates, and at our period of civilisation, should be so markedly prevalent. Our fogs and depressing atmospheres are, we are convinced, the fruitful originators of many of the severest forms of headache. On the subject of remedies, Dr. Brunton speaks with much confidence of antipyrin. Many medical men and their patients have expressed disappointment with the results of the administration of antipyrin. The cause of their disappointment we are assured is the defectiveness of their physiological knowledge. When once the headache has obtained a thorough grip of the patient his stomach ceases to secrete gastric juice, or to absorb either food or medicine. This is true and obvious in the case of bilious headaches, but not of all headaches. Absorption being entirely arrested, it is clear that whatever drug we give by the mouth there can be no physiological action, because it lies like a stone, unabsorbed in the stomach. Distressing headaches at this stage can, therefore, only be successfully treated by subcutaneous injections. There is, however, one very obvious moral here, or rather two. The first is that sufferers from headache should have their eyes promptly dealt with; and the second that the moment they are threatened with headache, and long before the pain comes on, they should resort to a proved remedy, antipyrin or another, in order that it may be well absorbed before arrest of absorption sets in.

### Rest the Recuperator.

The man who does not do his work with verve never does it really well; and the man who does not stick to it until success is victoriously achieved, is not made of stuff of the first quality. With those who are worth anything, the task of life, however tough it may be, and however long, has got to be completed, as the Americans put it. In these times of manifold competition the task is pretty certain to be long. It follows, therefore, that the man who cannot "stay" cannot complete it. He will have to be written down among the failures. Is life worth living for a capable man if it is to be a failure in the long run? Hardly! If this be the general feeling, it is obvious that "recuperators" of energy and capacity become of great importance to the man who is minded to avoid the paralysis of his career. Of all the recuperators of intellectual energy and freshness, there is one which is chief and has no second. That recuperator is rest. Let him who questions the superlative value of rest, try to do without the rest of sleep for a single week. Rest, to produce its full result, must be absolute; not merely the cessation of work, but the abandonment of care; the laying aside of responsibility also, as of a coat which is not to be worn for a period. The man whose brain is very tired must give his body rest as well as his intellect. A weary brain will not supply the muscles with energy for long walks or fatiguing toils. A large sofa in a large and airy room for a lounge in the winter, with two or three short and easy walks in the fresh air, is the ideal to be sought after: in the summer a hammock, in a quiet corner of the orchard or coppice, where the breezes are gentle, and the rustling of the leaves is soft. A mild and very occasional smoke for those who like it, and a tame novel to read for a few minutes at a time, three or four times a day, may help the sense of quiet and repose. Two, or three, or four weeks spent in this way will make any fairly healthy man young again, however worn out he may be. The intellectual worker should have two such seasons of complete rest every year. The freshness of his work would soon show the soundness of his philosophy.

### The Metropolitan Hospital Saturday Fund.

If elaborate care and close attention to detail could make this Fund a success Mr. Acland and Mr. Bunn would have achieved it long ago. It lacks, however, that touch of nature which makes the whole world kin. This is made very apparent by the able address of the Chairman, which deals with many subjects. A careful perusal has convinced us that the writer fails to grasp the importance of winning the sympathy not only of the working classes, but of all classes, for the Hospital Saturday movement. There is good sense but a lack of statesmanship about the address, which is abundantly noticeable in the paragraphs which deal with (1) the Provident Association, and (2) the existing hospitals. In commenting on the first, Mr. Acland ignores the teachings of past experience in regard to this movement, and in dealing with the existing hospitals, he makes no distinction between the worthy and the disreputable. He, unfortunately for the Fund, includes all hospitals and kindred institutions in one category, and so fails to do justice to the best. No chairman of a Hospital Saturday Fund can afford to take up such a position. We are sure it has been assumed by Mr. Acland from a want of appreciation of the true circumstances of the present situation, and we have no doubt that he will take an early opportunity to correct the mistake into which he has been led. What the Hospital Saturday Fund needs most is a quickening spirit which shall weld together hospital managers and the managers of the Hospital Saturday Fund, so that they may, through the District and Hospital Committees co-operate to secure adequate support for all the hospitals throughout London. At the present time we believe the work in the office of the Hospital Saturday Fund to be most efficiently done. The staff is intelligent and capable, the organisation is complete and adequate, and the constitution is sound. In these circumstances the Chairman should take heart, and by manifesting a wise and liberal spirit secure the goodwill and support of those without whose aid this Fund can never exercise any real power for good. At present, like its monthly journal, the fund lacks shape and powers of attraction. Let the journal be made into an eight-page paper of reasonable proportions, and the Fund quickened by the infusion of a kindly spirit, not only towards the working classes, but towards the hospitals, and all who have to do with hospitals and the treatment of the sick. Then, but not till then, it may prosper and increase.

